# Supplementary material for: The Impact of Sea Ice Cover on Microbial Communities in Antarctic Shelf Sediments
Source: Microorganisms. 2023 Jun 14;11(6):1572. doi: 10.3390/microorganisms11061572 (PMC10305693; doi:10.3390/microorganisms11061572)
Supplement: Supplementary file 1 [file microorganisms-11-01572-s001.zip › microorganisms-2320056-supplementary.pdf]

Supplementary Material

The impact of sea ice cover on microbial communities in Antarctic shelf sediments

Marwa Baloza [1,2,*], Susann Henkel [1], Sabine Kasten [1,3], Moritz Holtappels [1], Massimiliano Molari [4]

[1] Alfred Wegener Institute Helmholtz Centre for Polar and Marine Research, Am Handelshafen 12, 27570 Bremerhaven, Germany

[2] Faculty 2 Biology / Chemistry, University of Bremen, Leobener Str., Bremen, Germany

[3] Faculty of Geosciences, University of Bremen, Klagenfurter Str., Bremen, Germany

[4] HGF-MPG Joint Research Group for Deep-Sea Ecology and Technology, Max Planck Institute for Marine Microbiology, Bremen, Germany

*Correspondence: marwa.baloza@awi.de

Table S1: Overview of sampled stations during RV Polarstern expedition PS118. The table consists of sampling information for each station, number of sequences in each step of the bioinformatics workflow as well as alpha diversity indices: 1) observed richness, 2) Shannon and 3) inverse simpson, conducted using the R package 'Phyloseq'.

PANGAEA Station ID	Core ID	Sample ID	Latitude °S	Longitude ° W	Water depth (m)	Sampling Date	Sediment depth (cm)	No. of raw amplicons	No. of amplicons after QC and merging	Final no. of amplicons after removal of abs. singletons	Observed richness	Shanon index	Inverse simpson
PS118_5-3	St1-MUC1-C3-D1	St1_MUC1_C3_D1	64.983	57.753	428.3	04/03/2019	0-1_cm	140887	100733	78929	2676	7.2	593.0
PS118_5-3	St1-MUC1-C3-D2	St1_MUC1_C3_D2	64.983	57.753	428.3	04/03/2019	1-2_cm	142144	99595	77998	2854	7.3	748.1
PS118_5-3	St1-MUC1-C3-D3	St1_MUC1_C3_D3	64.983	57.753	428.3	04/03/2019	2-3_cm	123410	84167	66523	2540	7.2	693.9
PS118_5-3	St1-MUC1-C3-D4	St1_MUC1_C3_D4	64.983	57.753	428.3	04/03/2019	3-5_cm	33747	19921	16402	867	6.3	316.4
PS118_5-3	St1-MUC1-C3-D5	St1_MUC1_C3_D5	64.983	57.753	428.3	04/03/2019	5-7_cm	91310	61786	48871	1955	6.9	352.0
PS118_5-3	St1-MUC1-C3-D6	St1_MUC1_C3_D6	64.983	57.753	428.3	04/03/2019	14-16_cm	179202	134500	95356	2619	6.9	277.2
PS118_5-4	St1-MUC2-C2-D1	St1_MUC2_C2_D1	64.98	57.746	429	04/03/2019	0-1_cm	110346	78493	60313	2359	7.2	685.4
PS118_5-4	St1-MUC2-C2-D2	St1_MUC2_C2_D2	64.98	57.746	429	04/03/2019	1-2_cm	174794	115144	89945	2908	7.4	782.6
PS118_5-4	St1-MUC2-C2-D3	St1_MUC2_C2_D3	64.98	57.746	429	04/03/2019	2-3_cm	74508	51164	42254	1689	6.8	439.5
PS118_5-4	St1-MUC2-C2-D4	St1_MUC2_C2_D4	64.98	57.746	429	04/03/2019	3-5_cm	74457	24136	18998	644	5.9	164.2
PS118_5-4	St1-MUC2-C2-D5	St1_MUC2_C2_D5	64.98	57.746	429	04/03/2019	5-7_cm	63803	47087	27997	1174	6.3	146.4
PS118_5-4	St1-MUC2-C2-D6	St1_MUC2_C2_D6	64.98	57.746	429	04/03/2019	14-16_cm	111534	80429	56687	2009	6.8	371.3
PS118_5-4	St1-MUC2-C3-D1	St1_MUC2_C3_D1	64.98	57.746	429	04/03/2019	0-1_cm	301878	229827	178738	4659	7.6	795.1
PS118_5-4	St1-MUC2-C3-D2	St1_MUC2_C3_D2	64.98	57.746	429	04/03/2019	1-2_cm	226974	170001	134187	3942	7.5	784.3
PS118_5-4	St1-MUC2-C3-D3	St1_MUC2_C3_D3	64.98	57.746	429	04/03/2019	2-3_cm	199544	144578	116519	3152	7.3	568.2
PS118_5-4	St1-MUC2-C3-D4	St1_MUC2_C3_D4	64.98	57.746	429	04/03/2019	3-5_cm	130948	97863	75485	2492	7.1	375.6
PS118_5-4	St1-MUC2-C3-D5	St1_MUC2_C3_D5	64.98	57.746	429	04/03/2019	5-7_cm	348405	277222	179089	3962	7.3	277.1

PS118_5-4	St1-MUC2-C3-D6	St1_MUC2_C3_D6	64.98	57.746	429	04/03/2019	14-16_cm	96189	70140	52936	1696	6.5	210.6
PS118_8-5	St2-MUC1-C3-D1	St2_MUC1_C3_D1	63.971	55.905	413	11/03/2019	0-1_cm	177957	151741	45257	1333	6.3	196.7
PS118_8-5	PS118-2-MUC1-C3-D2	St2_MUC1_C3_D2	63.971	55.905	413	11/03/2019	1-2_cm	99390	85901	13410	516	5.5	111.3
PS118_8-5	PS118-2-MUC1-C3-D3	St2_MUC1_C3_D3	63.971	55.905	413	11/03/2019	2-3_cm	167308	132261	50070	1477	6.3	153.6
PS118_8-5	PS118-2-MUC1-C3-D4	St2_MUC1_C3_D4	63.971	55.905	413	11/03/2019	3-5_cm	143912	121483	25115	778	5.6	99.9
PS118_8-5	PS118-2-MUC1-C3-D5	St2_MUC1_C3_D5	63.971	55.905	413	11/03/2019	5-7_cm	65553	53080	20397	883	6.1	227.5
PS118_8-5	PS118-2-MUC1-C3-D6	St2_MUC1_C3_D6	63.971	55.905	413	11/03/2019	14-16_cm	73741	58695	30629	885	5.8	134.4
PS118_8-6	PS118-2-MUC2-C3-D1	St2_MUC2_C3_D1	63.968	55.906	415	11/03/2019	0-1_cm	29332	22671	5743	300	5.1	79.2
PS118_8-6	PS118-2-MUC2-C3-D2	St2_MUC2_C3_D2	63.968	55.906	415	11/03/2019	1-2_cm	120550	98001	30162	1113	6.3	247.2
PS118_8-6	PS118-2-MUC2-C3-D3	St2_MUC2_C3_D3	63.968	55.906	415	11/03/2019	2-3_cm	63915	42981	34347	1189	6.5	357.3
PS118_8-6	PS118-2-MUC2-C3-D4	St2_MUC2_C3_D4	63.968	55.906	415	11/03/2019	3-5_cm	30633	18579	13158	482	5.5	109.6
PS118_8-6	PS118-2-MUC2-C3-D5	St2_MUC2_C3_D5	63.968	55.906	415	11/03/2019	5-7_cm	62333	37705	31078	1367	6.7	482.2
PS118_8-6	PS118-2-MUC2-C3-D6	St2_MUC2_C3_D6	63.968	55.906	415	11/03/2019	14-16_cm	122012	89107	49363	1836	6.8	395.3
PS118_8-7	PS118-2-MUC3-C2-D1	St2_MUC3_C2_D1	63.965	55.908	415	11/03/2019	0-1_cm	104073	71432	38974	1448	6.5	251.1
PS118_8-7	PS118-2-MUC3-C2-D2	St2_MUC3_C2_D2	63.965	55.908	415	11/03/2019	1-2_cm	230837	182367	102855	2139	6.2	81.4
PS118_8-7	PS118-2-MUC3-C2-D3	St2_MUC3_C2_D3	63.965	55.908	415	11/03/2019	2-3_cm	72603	58387	20039	763	6.0	200.3
PS118_8-7	PS118-2-MUC3-C2-D4	St2_MUC3_C2_D4	63.965	55.908	415	11/03/2019	3-5_cm	59829	37896	30171	919	6.1	178.3
PS118_8-7	PS118-2-MUC3-C2-D5	St2_MUC3_C2_D5	63.965	55.908	415	11/03/2019	5-7_cm	95488	59253	48605	1911	7.0	578.4
PS118_8-7	PS118-2-MUC3-C2-D6	St2_MUC3_C2_D6	63.965	55.908	415	11/03/2019	14-16_cm	50549	33348	21217	1093	6.5	372.6
PS118_12-3	PS118-3-MUC2-C3-D1	St3_MUC2_C3_D1	63.812	55.731	454	14/03/2019	0-1_cm	453364	385371	99819	2166	6.3	124.9
PS118_12-3	PS118-3-MUC2-C3-D2	St3_MUC2_C3_D2	63.812	55.731	454	14/03/2019	1-2_cm	62135	49578	18299	601	5.5	89.4
PS118_12-3	PS118-3-MUC2-C3-D3	St3_MUC2_C3_D3	63.812	55.731	454	14/03/2019	2-3_cm	656174	547093	140178	2488	6.4	118.9
PS118_12-3	PS118-3-MUC2-C3-D4	St3_MUC2_C3_D4	63.812	55.731	454	14/03/2019	3-5_cm	57424	34989	28350	1346	6.7	525.2
PS118_12-3	PS118-3-MUC2-C3-D5	St3_MUC2_C3_D5	63.812	55.731	454	14/03/2019	5-7_cm	145261	104840	51278	1847	6.8	375.2
PS118_12-3	PS118-3-MUC2-C3-D6	St3_MUC2_C3_D6	63.812	55.731	454	14/03/2019	14-16_cm	63868	54132	9638	399	5.3	96.6
PS118_12-3	PS118-3-MUC2-C5-D1	St3_MUC2_C4_D1	63.812	55.731	454	14/03/2019	0-1_cm	300089	245619	96490	2250	6.8	328.7
PS118_12-3	PS118-3-MUC2-C5-D2	St3_MUC2_C4_D2	63.812	55.731	454	14/03/2019	1-2_cm	59559	47925	21189	714	5.6	102.5
PS118_12-3	PS118-3-MUC2-C5-D3	St3_MUC2_C4_D3	63.812	55.731	454	14/03/2019	2-3_cm	195404	158000	72449	1640	6.4	245.5
PS118_12-3	PS118-3-MUC2-C5-D4	St3_MUC2_C4_D4	63.812	55.731	454	14/03/2019	3-5_cm	77992	58391	45989	968	5.8	118.1
PS118_12-3	PS118-3-MUC2-C5-D5	St3_MUC2_C4_D5	63.812	55.731	454	14/03/2019	5-7_cm	97308	63068	50631	1906	7.0	529.3
PS118_12-3	PS118-3-MUC2-C5-D6	St3_MUC2_C4_D6	63.812	55.731	454	14/03/2019	14-16_cm	52684	31612	24373	1246	6.6	433.9
PS118_12-5	PS118-3-MUC4-C2-D1	St3_MUC4_C2_D1	63.814	55.712	454	14/03/2019	0-1_cm	751311	612485	250824	3841	6.8	223.8
PS118_12-5	PS118-3-MUC4-C2-D2	St3_MUC4_C2_D2	63.814	55.712	454	14/03/2019	1-2_cm	121307	97019	46493	1143	5.9	134.7
PS118_12-5	PS118-3-MUC4-C2-D3	St3_MUC4_C2_D3	63.814	55.712	454	14/03/2019	2-3_cm	72544	61335	7815	331	5.0	58.8
PS118_12-5	PS118-3-MUC4-C2-D4	St3_MUC4_C2_D4	63.814	55.712	454	14/03/2019	3-5_cm	32559	18324	15098	835	6.3	338.0
PS118_12-5	PS118-3-MUC4-C2-D5	St3_MUC4_C2_D5	63.814	55.712	454	14/03/2019	5-7_cm	80398	52614	33238	1228	6.3	202.5
PS118_12-5	PS118-3-MUC4-C2-D6	St3_MUC4_C2_D6	63.814	55.712	454	14/03/2019	14-16_cm	30286	26608	942	65	3.7	29.0
PS118_13-5	PS118-4-MUC3-C3-D1	St4_MUC3_C3_D1	63.054	54.316	440	17/03/2019	0-1_cm	77488	60941	20473	828	6.0	191.8
PS118_13-5	PS118-4-MUC3-C3-D2	St4_MUC3_C3_D2	63.054	54.316	440	17/03/2019	1-2_cm	71350	57547	17397	646	5.6	115.6
PS118_13-5	PS118-4-MUC3-C3-D3	St4_MUC3_C3_D3	63.054	54.316	440	17/03/2019	2-3_cm	189790	156956	57731	1181	6.0	152.4
PS118_13-5	PS118-4-MUC3-C3-D4	St4_MUC3_C3_D4	63.054	54.316	440	17/03/2019	3-5_cm	32597	20243	17026	795	6.2	327.6
PS118_13-5	PS118-4-MUC3-C3-D5	St4_MUC3_C3_D5	63.054	54.316	440	17/03/2019	5-7_cm	117420	78329	57409	1738	6.7	255.0
PS118_13-5	PS118-4-MUC3-C3-D6	St4_MUC3_C3_D6	63.054	54.316	440	17/03/2019	14-16_cm	41248	22766	17485	1000	6.4	389.9

PS118_13-5	PS118-4-MUC3-C4-D1	St4_MUC3_C4_D1	63.054	54.316	440	17/03/2019	0-1_cm	52717	40060	12139	540	5.7	160.1
PS118_13-5	PS118-4-MUC3-C4-D2	St4_MUC3_C4_D2	63.054	54.316	440	17/03/2019	1-2_cm	146061	118378	39301	1186	6.3	237.0
PS118_13-5	PS118-4-MUC3-C4-D3	St4_MUC3_C4_D3	63.054	54.316	440	17/03/2019	2-3_cm	515887	438770	105635	2189	6.6	261.7
PS118_13-5	PS118-4-MUC3-C4-D4	St4_MUC3_C4_D4	63.054	54.316	440	17/03/2019	3-5_cm	48086	28084	22636	1062	6.4	333.7
PS118_13-5	PS118-4-MUC3-C4-D5	St4_MUC3_C4_D5	63.054	54.316	440	17/03/2019	5-7_cm	54466	30371	22280	1195	6.6	425.5
PS118_13-5	PS118-4-MUC3-C4-D6	St4_MUC3_C4_D6	63.054	54.316	440	17/03/2019	14-16_cm	47878	39822	7623	307	4.9	55.5
PS118_13-5	PS118-4-MUC3-C5-D1	St4_MUC3_C5_D1	63.054	54.316	440	17/03/2019	0-1_cm	693693	550245	205124	3489	6.9	300.2
PS118_13-5	PS118-4-MUC3-C5-D2	St4_MUC3_C5_D2	63.054	54.316	440	17/03/2019	1-2_cm	66669	47607	31600	1081	6.1	171.2
PS118_13-5	PS118-4-MUC3-C5-D3	St4_MUC3_C5_D3	63.054	54.316	440	17/03/2019	2-3_cm	79110	65747	21396	697	5.6	110.2
PS118_13-5	PS118-4-MUC3-C5-D4	St4_MUC3_C5_D4	63.054	54.316	440	17/03/2019	3-5_cm	44097	29097	24954	1067	6.5	376.8
PS118_13-5	PS118-4-MUC3-C5-D5	St4_MUC3_C5_D5	63.054	54.316	440	17/03/2019	5-7_cm	67846	44010	28515	1194	6.4	240.5
PS118_13-5	PS118-4-MUC3-C5-D6	St4_MUC3_C5_D6	63.054	54.316	440	17/03/2019	14-16_cm	26018	14592	10329	591	5.9	225.5
PS118_62-2	PS118-7-MUC1-C6-D1	St7_MUC1_C6_D1	60.934	46.559	329	28/03/2019	0-1_cm	275384	237585	48709	1191	5.8	95.8
PS118_62-2	PS118-7-MUC1-C6-D2	St7_MUC1_C6_D2	60.934	46.559	329	28/03/2019	1-2_cm	181518	144729	46267	1403	6.4	228.7
PS118_62-2	PS118-7-MUC1-C6-D3	St7_MUC1_C6_D3	60.934	46.559	329	28/03/2019	2-3_cm	44761	28363	22822	1031	6.4	367.9
PS118_62-2	PS118-7-MUC1-C6-D4	St7_MUC1_C6_D4	60.934	46.559	329	28/03/2019	3-5_cm	40195	25580	15579	865	6.2	259.6
PS118_62-2	PS118-7-MUC1-C6-D5	St7_MUC1_C6_D5	60.934	46.559	329	28/03/2019	5-7_cm	28882	15644	12154	683	6.0	228.4
PS118_62-2	PS118-7-MUC1-C6-D6	St7_MUC1_C6_D6	60.934	46.559	329	28/03/2019	14-16_cm	49297	33144	13475	732	6.0	214.2
PS118_62-2	PS118-7-MUC1-C7-D1	St7_MUC1_C7_D1	60.934	46.559	329	28/03/2019	0-1_cm	510810	424245	110023	2013	6.3	163.9
PS118_62-2	PS118-7-MUC1-C7-D2	St7_MUC1_C7_D2	60.934	46.559	329	28/03/2019	1-2_cm	55283	43902	26043	675	5.5	102.7
PS118_62-2	PS118-7-MUC1-C7-D3	St7_MUC1_C7_D3	60.934	46.559	329	28/03/2019	2-3_cm	73160	60191	18535	755	6.0	224.2
PS118_62-2	PS118-7-MUC1-C7-D4	St7_MUC1_C7_D4	60.934	46.559	329	28/03/2019	3-5_cm	49056	32078	26207	1167	6.6	415.6
PS118_62-2	PS118-7-MUC1-C7-D5	St7_MUC1_C7_D5	60.934	46.559	329	28/03/2019	5-7_cm	51510	32817	20328	960	6.3	293.2
PS118_62-2	PS118-7-MUC1-C7-D6	St7_MUC1_C7_D6	60.934	46.559	329	28/03/2019	14-16_cm	19051	16370	16321	12	1.0	1.8
PS118_62-2	PS118-7-MUC1-C8-D1	St7_MUC1_C8_D1	60.934	46.559	329	28/03/2019	0-1_cm	107808	90441	20830	562	5.2	56.4
PS118_62-2	PS118-7-MUC1-C8-D2	St7_MUC1_C8_D2	60.934	46.559	329	28/03/2019	1-2_cm	97399	82141	11493	471	5.4	92.9
PS118_62-2	PS118-7-MUC1-C8-D3	St7_MUC1_C8_D3	60.934	46.559	329	28/03/2019	2-3_cm	48768	31095	24791	924	6.3	317.9
PS118_62-2	PS118-7-MUC1-C8-D4	St7_MUC1_C8_D4	60.934	46.559	329	28/03/2019	3-5_cm	18057	3718	3109	173	4.9	112.5
PS118_62-2	PS118-7-MUC1-C8-D5	St7_MUC1_C8_D5	60.934	46.559	329	28/03/2019	5-7_cm	38104	21983	16390	815	6.1	214.1
PS118_62-2	PS118-7-MUC1-C8-D6	St7_MUC1_C8_D6	60.934	46.559	329	28/03/2019	14-16_cm	108788	82819	39026	1352	6.4	213.6
Total								11874014	9141087	4252065			

Table S2: Output PERMANOVA and distance-based redundancy analysis (dbRDA).

		Df	SumsOfSqs	F	R^2	Pr(>F)
adonis: (data.clr [c(St1.MUC1.C3.D1, St1.MUC2.C2.D1, St1.MUC2.C3.D1, St2.MUC1.C3.D1, PS118.2.MUC2.C3.D1, PS118.2.MUC3.C2.D1, PS118.3.MUC2.C3.D1, PS118.3.MUC2.C5.D1, PS118.3.MUC4.C2.D1, PS118.4.MUC3.C3.D1, PS118.4.MUC3.C4.D1, PS118.4.MUC3.C5.D1, PS118.7.MUC1.C6.D1, PS118.7.MUC1.C7.D1, PS118.7.MUC1.C8.D1),]~ Sea_Ice_index, data = Env_data, method = 'euclidean', permutations = 9999)	Sea ice index	1	3794	1.8916	0.12702	0.0264
	Residuals	13	26074		0.87298	
	Total	14	29868		1.00000	
adonis: (data.clr ~ Sea_Ice_index/ Layer, data = Env_data, strata = Env_data$Core_ID, method = 'euclidean', permutations = 9999)	Sea ice index	1	5827	4.4165	0.04590	0.0001
	Sea ice index: layer	5	11604	1.7590	0.09141	0.0001
	Residuals	83	109509		0.86268	
	Total	89	126941		1.00000	
dbrda: data.clr ~ C/N ratio + O_2 (µmol/L) + Fe(II) (µmol/L) + Fe (III)/Al + S/Al + Sulfat (mmol/L) + DIC(µmol/L)	**ANOVA**	**Df**	**Variance**	**F**	**Pr(>F)**	**p.adjust**
	C/N ratio	1	25.98	1.8687	0.008	0.011
	O_2 (µmol/L)	1	52.19	3.7547	0.001	0.003
	Fe(II) (µmol/L)	1	51.49	3.7039	0.001	0.003
	Fe (III)/Al	1	27.93	2.0094	0.003	0.005
	S/Al	1	30.45	2.1903	0.003	0.005
	Sulfate (mmol/L)	1	13.27	0.9546	0.476	0.476
	DIC(µmol/L)	1	22.74	1.6361	0.022	0.025
	Residual	82	1139.83			

Table S3: ASVs highly abundant in the ferruginous zone. Differential abundance of taxa between surface sediments (0-3 cm) of stations Shelf St1 and Shelf St4 samples according to the t-test based metastats

analysis. Differentially abundant taxa having statistically significant differences for parametric test (glm.eBH) (p<0.01) and non-parametric test (kw.ep) (p<0.05) are reported for each ASV per sample.

	kw.ep	glm.eBH	St4. MUC3.C5.D3	St4. MUC3.C5.D2	St4. MUC3.C5.D1	St4. MUC3.C4.D3	St4. MUC3.C4.D2	St4. MUC3.C4.D1	St4. MUC3.C3.D3	St4. MUC3.C3.D2	St4. MUC3.C3.D1
asv2	0.0017	0.0005	5.87	1.88	2.57	2.72	1.96	2.89	3.54	4.60	3.86
asv4	0.0003	0.0002	2.39	0.53	1.83	1.10	1.73	1.41	2.36	1.58	2.10
asv15	0.0003	0.0007	2.00	0.48	0.61	0.42	0.42	0.43	1.75	1.32	0.92
asv5	0.0003	0.0000	1.66	0.60	1.26	1.06	1.34	1.20	1.23	1.87	1.93
asv18	0.0003	0.0001	1.99	2.01	0.21	0.25	0.22	0.20	1.67	1.90	0.26
asv26	0.0003	0.0000	1.30	0.11	0.47	0.42	0.52	1.23	2.10	0.31	0.60
asv16	0.0004	0.0000	1.76	0.95	0.54	0.38	0.51	0.56	1.46	1.21	0.60
asv6	0.0015	0.0009	1.52	2.03	0.76	0.60	0.66	0.82	1.45	2.06	0.89
asv21	0.0003	0.0004	1.22	0.17	0.35	0.32	0.54	1.04	1.94	0.34	0.55
asv13	0.0003	0.0000	1.27	0.20	0.77	0.56	1.27	0.79	1.29	0.59	1.17
asv42	0.0004	0.0000	1.06	0.42	0.22	0.21	0.28	0.21	1.64	0.96	0.24
asv23	0.0004	0.0049	0.87	0.08	0.22	0.10	0.66	3.59	1.83	0.15	0.29
asv12	0.0003	0.0001	0.84	0.19	1.31	0.97	0.63	1.12	0.72	0.46	1.25
asv14	0.0044	0.0034	1.08	0.38	0.72	0.51	0.78	0.36	0.89	0.80	0.96
asv24	0.0003	0.0006	0.85	0.38	0.65	0.37	0.54	0.39	0.99	0.63	0.63
asv31	0.0003	0.0000	0.84	0.42	0.23	0.32	0.47	0.35	0.96	0.72	0.33
asv20	0.0016	0.0013	1.20	0.18	0.54	0.17	1.34	1.02	0.75	0.34	0.90
asv8	0.0006	0.0000	1.04	0.66	1.11	0.56	0.41	0.19	0.50	1.80	1.05
asv7	0.0003	0.0001	0.97	3.20	0.48	0.46	0.10	0.26	0.65	2.02	0.39
asv9	0.0003	0.0000	1.09	1.95	1.20	0.61	0.27	0.27	0.31	2.35	1.10
asv41	0.0003	0.0008	0.68	0.19	0.64	0.32	0.62	0.29	0.69	0.54	0.86
asv19	0.0020	0.0038	0.83	0.58	0.76	0.45	0.17	0.00	0.32	1.41	0.72
asv40	0.0003	0.0003	0.76	0.34	0.54	0.30	0.32	0.32	0.43	0.63	0.75
asv11	0.0005	0.0010	0.66	0.77	1.45	0.53	0.28	0.17	0.16	1.22	1.16
asv68	0.0003	0.0001	0.66	0.09	0.20	0.40	0.17	0.17	0.29	0.36	0.34
asv38	0.0003	0.0000	0.51	0.41	0.41	0.26	0.28	0.28	0.47	0.46	0.52
asv61	0.0035	0.0012	0.33	0.06	0.39	0.34	0.48	0.52	0.43	0.39	0.59
asv60	0.0037	0.0031	0.45	0.09	0.27	0.16	0.50	0.38	0.44	0.27	0.54
asv106	0.0016	0.0013	0.49	0.67	0.16	0.13	0.13	0.00	0.41	0.74	0.28
asv58	0.0004	0.0004	0.38	0.27	0.30	0.23	0.23	0.12	0.42	0.37	0.40
asv54	0.0051	0.0041	0.41	0.38	0.16	0.14	0.24	0.16	0.45	0.32	0.14
asv82	0.0003	0.0000	0.43	0.11	0.25	0.23	0.31	0.18	0.34	0.39	0.31
asv96	0.0003	0.0000	0.36	0.24	0.21	0.17	0.18	0.19	0.41	0.29	0.38
asv129	0.0003	0.0000	0.41	0.09	0.14	0.10	0.28	0.26	0.43	0.34	0.20
asv56	0.0037	0.0066	0.22	0.32	0.33	0.61	0.07	0.07	0.07	0.21	0.27
asv59	0.0015	0.0016	0.55	0.73	0.34	0.18	0.04	0.00	0.15	0.68	0.25
asv50	0.0019	0.0011	0.36	0.22	0.80	0.50	0.13	0.15	0.00	0.61	0.59
asv126	0.0003	0.0000	0.34	0.16	0.21	0.20	0.19	0.17	0.29	0.14	0.23
asv103	0.0016	0.0014	0.32	0.00	0.27	0.34	0.10	0.72	0.13	0.10	0.36

asv174	0.0003	0.0000	0.41	0.23	0.05	0.06	0.08	0.13	0.32	0.26	0.12
asv51	0.0005	0.0034	0.28	0.32	0.38	0.37	0.19	0.43	0.14	0.46	0.46
asv27	0.0023	0.0015	0.37	0.80	0.36	0.25	0.16	0.29	0.16	1.31	0.39
asv64	0.0003	0.0000	0.37	0.13	0.27	0.19	0.13	0.33	0.19	0.39	0.21
asv69	0.0003	0.0000	0.12	0.17	0.59	0.52	0.30	0.14	0.11	0.34	0.70
asv79	0.0003	0.0000	0.39	0.10	0.15	0.07	0.56	0.68	0.27	0.32	0.26
asv78	0.0018	0.0034	0.10	0.22	0.56	0.54	0.33	0.00	0.06	0.07	0.76
asv77	0.0003	0.0000	0.34	0.09	0.55	0.30	0.10	0.14	0.05	0.17	0.43
asv191	0.0003	0.0000	0.21	0.04	0.13	0.07	0.24	0.19	0.41	0.09	0.18
asv197	0.0003	0.0002	0.23	0.04	0.10	0.09	0.21	0.17	0.35	0.11	0.16
asv119	0.0017	0.0007	0.22	0.17	0.22	0.13	0.17	0.00	0.31	0.25	0.20

	kw.ep	glm.eBH	St1. MUC1.C3.D1	St1. MUC1.C3.D2	St1. MUC1.C3.D3	St1. MUC2.C2.D1	St1. MUC2.C2.D2	St1. MUC2.C2.D3	St1. MUC2.C3.D1	St1. MUC2.C3.D2	St1. MUC2.C3.D3
asv2	0.0017	0.0005	0.24	0.33	0.54	0.64	0.96	1.91	0.45	1.12	1.73
asv4	0.0003	0.0002	0.00	0.00	0.00	0.00	0.06	0.09	0.02	0.07	0.12
asv15	0.0003	0.0007	0.00	0.00	0.00	0.00	0.01	0.07	0.01	0.01	0.08
asv5	0.0003	0.0000	0.03	0.01	0.04	0.00	0.04	0.02	0.02	0.02	0.03
asv18	0.0003	0.0001	0.00	0.00	0.00	0.00	0.00	0.03	0.00	0.01	0.01
asv26	0.0003	0.0000	0.00	0.00	0.00	0.00	0.00	0.00	0.01	0.01	0.00
asv16	0.0004	0.0000	0.08	0.07	0.11	0.08	0.11	0.20	0.09	0.11	0.17
asv6	0.0015	0.0009	0.22	0.27	0.28	0.27	0.29	0.52	0.34	0.37	0.37
asv21	0.0003	0.0004	0.00	0.00	0.00	0.00	0.01	0.07	0.02	0.04	0.05
asv13	0.0003	0.0000	0.05	0.08	0.06	0.00	0.06	0.11	0.06	0.08	0.08
asv42	0.0004	0.0000	0.04	0.03	0.01	0.04	0.03	0.01	0.03	0.04	0.03
asv23	0.0004	0.0049	0.03	0.00	0.00	0.03	0.00	0.02	0.03	0.03	0.02
asv12	0.0003	0.0001	0.00	0.00	0.00	0.00	0.00	0.00	0.00	0.00	0.00
asv14	0.0044	0.0034	0.10	0.15	0.19	0.15	0.21	0.28	0.17	0.23	0.27
asv24	0.0003	0.0006	0.00	0.00	0.00	0.00	0.00	0.05	0.01	0.03	0.04
asv31	0.0003	0.0000	0.00	0.00	0.00	0.00	0.00	0.00	0.00	0.00	0.00
asv20	0.0016	0.0013	0.14	0.09	0.05	0.11	0.09	0.08	0.18	0.12	0.09
asv8	0.0006	0.0000	0.01	0.02	0.01	0.02	0.05	0.11	0.02	0.06	0.10
asv7	0.0003	0.0001	0.00	0.00	0.00	0.00	0.00	0.06	0.01	0.01	0.03
asv9	0.0003	0.0000	0.00	0.00	0.00	0.00	0.00	0.00	0.00	0.00	0.00
asv41	0.0003	0.0008	0.00	0.00	0.00	0.00	0.02	0.05	0.01	0.03	0.04
asv19	0.0020	0.0038	0.00	0.00	0.00	0.00	0.00	0.04	0.00	0.01	0.02
asv40	0.0003	0.0003	0.00	0.00	0.00	0.00	0.00	0.00	0.00	0.02	0.03
asv11	0.0005	0.0010	0.00	0.01	0.00	0.00	0.00	0.05	0.01	0.03	0.07
asv68	0.0003	0.0001	0.00	0.00	0.00	0.00	0.01	0.02	0.00	0.00	0.00
asv38	0.0003	0.0000	0.00	0.00	0.00	0.00	0.00	0.00	0.00	0.00	0.00
asv61	0.0035	0.0012	0.08	0.04	0.06	0.05	0.05	0.06	0.06	0.07	0.08
asv60	0.0037	0.0031	0.07	0.06	0.07	0.08	0.08	0.09	0.08	0.10	0.12
asv106	0.0016	0.0013	0.00	0.00	0.00	0.00	0.00	0.00	0.00	0.00	0.00

asv58	0.0004	0.0004	0.00	0.00	0.00	0.00	0.01	0.04	0.01	0.02	0.02
asv54	0.0051	0.0041	0.06	0.05	0.06	0.09	0.09	0.11	0.08	0.08	0.08
asv82	0.0003	0.0000	0.00	0.00	0.00	0.00	0.00	0.00	0.00	0.00	0.00
asv96	0.0003	0.0000	0.00	0.00	0.00	0.00	0.00	0.00	0.00	0.00	0.00
asv129	0.0003	0.0000	0.00	0.00	0.00	0.00	0.00	0.00	0.00	0.00	0.00
asv56	0.0037	0.0066	0.03	0.03	0.04	0.02	0.04	0.06	0.02	0.02	0.05
asv59	0.0015	0.0016	0.00	0.00	0.00	0.00	0.00	0.06	0.00	0.00	0.02
asv50	0.0019	0.0011	0.00	0.00	0.00	0.00	0.00	0.00	0.00	0.00	0.00
asv126	0.0003	0.0000	0.00	0.00	0.00	0.00	0.00	0.00	0.00	0.00	0.00
asv103	0.0016	0.0014	0.00	0.00	0.00	0.00	0.00	0.00	0.00	0.00	0.00
asv174	0.0003	0.0000	0.00	0.00	0.00	0.00	0.00	0.00	0.00	0.00	0.00
asv51	0.0005	0.0034	0.09	0.13	0.08	0.08	0.09	0.15	0.12	0.09	0.08
asv27	0.0023	0.0015	0.04	0.06	0.06	0.08	0.08	0.28	0.06	0.11	0.20
asv64	0.0003	0.0000	0.00	0.00	0.00	0.00	0.00	0.00	0.00	0.00	0.00
asv69	0.0003	0.0000	0.00	0.00	0.00	0.00	0.00	0.04	0.00	0.00	0.00
asv79	0.0003	0.0000	0.00	0.00	0.00	0.00	0.00	0.00	0.00	0.00	0.00
asv78	0.0018	0.0034	0.00	0.00	0.00	0.00	0.00	0.00	0.00	0.00	0.00
asv77	0.0003	0.0000	0.00	0.00	0.00	0.00	0.00	0.00	0.00	0.00	0.00
asv191	0.0003	0.0000	0.00	0.00	0.00	0.00	0.00	0.00	0.00	0.00	0.00
asv197	0.0003	0.0002	0.00	0.00	0.00	0.00	0.00	0.00	0.00	0.00	0.00
asv119	0.0017	0.0007	0.00	0.00	0.00	0.00	0.00	0.00	0.00	0.00	0.00

Table S4: The most closely related sequences with identity (ID) > 99%, as identified with BLASTn (GeneBank nucleotide database, 12 June 2022).

ASV	GeneBank ID ≥ 99% similarity	Environment(s)
asv2	FN429801.1; AF530115.1; EU287191.1; KC631541.1; GU197468.1; JQ863508.1; GQ357030.1	Antarctic cold seep sediment; Antarctic shelf sediments; Arctic surface sediment; aquaculture farm sediment; intertidal sediment; suboxic marine sediment; methane seep sediment.
asv4	EU857895.1; FN396623.1; EU362314.1; KF440288.1; KX097349.1; FJ753062.1	Antarctic marine sediment; Arctic marine surface sediment; tidal flat sediment; mud volcano; hydrothermal deposits and seafloor sediments; suboxic sediment.
asv5	KJ566283.1; JF268353.1	Arctic marine sediment; deep-sea methane seep.
asv6	KY190828.1; EU287223.1; HQ191097.1; DQ351780.1; JQ863426.1; KC463739.1; KX097752.1; KF624175.1; FM179882.1; AB305481.1; KF799091.1; AJ880527.1	Antarctic marine sediment; Arctic marine sediment; muddy intertidal sediment; Adherent bacteria in heavy metal contaminated marine sediments; anoxic marine sediment; shallow-water hydrothermal vent sediments; deep-sea sediment; oxic pyrite-particle; methane seeps; hydrothermal sediments; Ciona intestinal gut; tidal flat sediments.
asv13	FN396637.1; KX172653.1; EU491847.1; KM203427.1; JX226991.1	Arctic marine surface sediment; deep sea marine sediment; seafloor lavas; deep-sea coral; deep-sea polymetallic nodules.
asv15	AF530125.1; GU292255.1; JF767462.1; GU996528.1; AJ704698.1; HQ703820.1; FJ873360.1	Antarctic continental shelf sediments; Arctic marine sediment; salmon farm marine sediment; crude oil alkanes; marine sediment from mud volcano; marine sediment; methane-rich cold seep.
asv16	KY190870.1; FJ223335.1; KY190827.1; FN396623.1; EU362314.1; KX097349.1; HE803925.1; KJ615939.1; FJ753062.1; KC631584.1; JQ579662.1	Antarctica marine sediment; Antarctica sea-bed hypoxia and sediment; Arctic marine surface sediment; mud volcano; tidal flat sediments; deep-sea sediment; marine seabed sediments; sediment with high Fe-Mn concretions; suboxic sediment; marine finfish aquaculture farm sediment; oil-polluted subtidal sediments .

asv 18	FN396617.1; MK175686.1; AB806723.1; JN621370.1; GQ246294.1; FJ873358.1; AM911597.1; FJ264600.1; KX088614.1; KX097659.1; KM356755.1; JQ036284.1; JQ863439.1;GQ357027.1; GQ261776.1; AB305506.1; MG002285.1; KF741490.1; HF922369.1; HE978803.1; JQ925095.1; JQ579936.1; DQ351760.1; KC631463.1; FJ752975.1	Arctic marine sediment; Coral microbiome; Ocean drilling core; oxide-rich marine sediments; marine sediments; methane-rich cold seep sediments; cold-water coral; methane seep sediment; Anaerobic oxidation of methane by coastal sediment; deep-sea sediment; Methane Seep Sediments; Methane Seep Carbonate Nodules; anoxic marine surface sediment; methane seep sediment; deep sea sediment; hydrothermal sediments; subsurface seawater; salt marsh sediment; high-pressure membrane capsule bioreactor; intertidal sediments; cold seep sediment; oil-polluted subtidal sediments; heavy metal contaminated marine sediments; marine finfish aquaculture farm sediment; ambient suboxic sediment.
asv21	EU050902.1; FJ223315.1; JQ197846.1; AY678530.1; AY171369.1	Arctic marine sediment; marine sediment coupling of sea-bed hypoxia; seawater next to dolphin; estuarine sediment; marine sediment.
asv23	AB598201.1; JF767423.1; KF799089.1	Sub-seafloor sediments; salmon farm marine sediment; gut microbiota.
asv26	FJ223340.1; EU287278.1; FJ659158.1; EU346635.1; MK224673.1 KX172349.1; GU584516.1; JQ200082.1; JX983867.1; GQ356980.1; AM072619.1	Antarctic marine sediment coupling of sea-bed hypoxia; Arctic surface sediment; aerobic anoxygenic photosynthesis associated with colonial ascidians; marine sponge; crustose coralline algae; seafloor sediment; marine hydrocarbon seeps; seawater; next to dolphin; biofilm; methane seep sediment; tidal-flat sediments.
asv42	KY190910.1; FJ223418.1; GU292246.1; MH929595.1;NR_152677.1; KM356330.1; KF268937.1; JQ436107.1; JQ661132.1; JQ580032.1; MW245850.1; NR042612.1; KF616716.1; FJ229466.1; DQ890444.1	Antarctic marine sediment; Antarctic sediment coupling of sea-bed hypoxia; Arctic Circle marine sediment; tundra soil; marine sediment; sediments and carbonates from the methane seep environments; marine sediment with Apatite and Chitin; seawater;

		soil; oil-polluted subtidal sediments; marine sponge; red algae; methane seeps; intertidal sand biofilm; mud shrimp.

Shelf St1

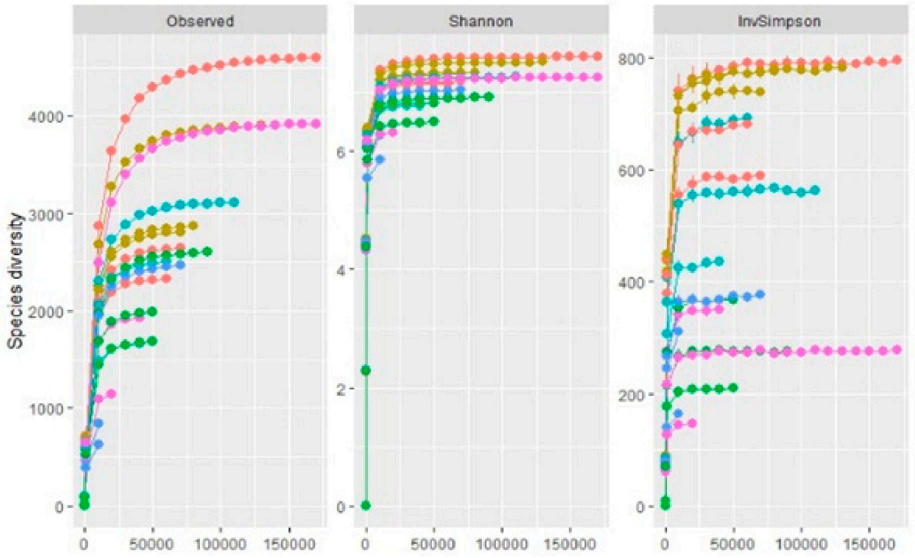

Shelf St2

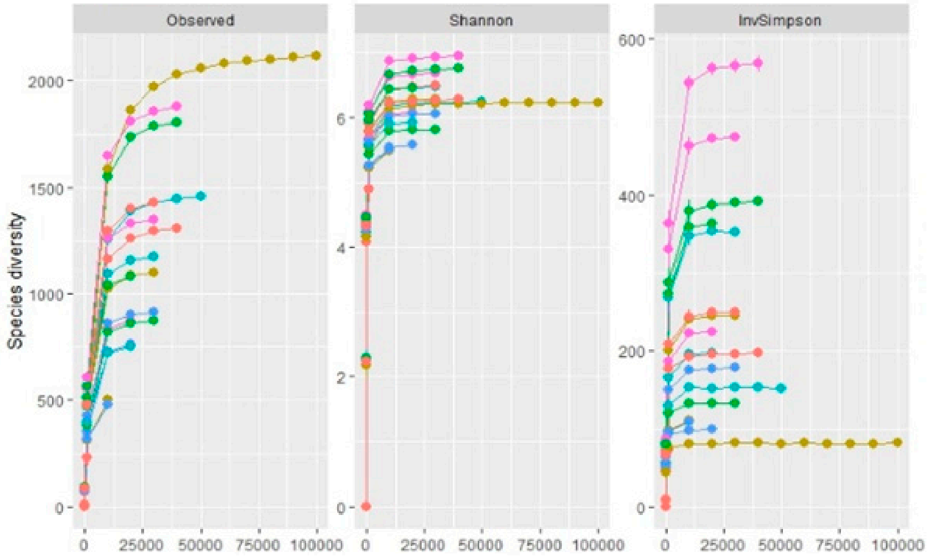

Shelf St3

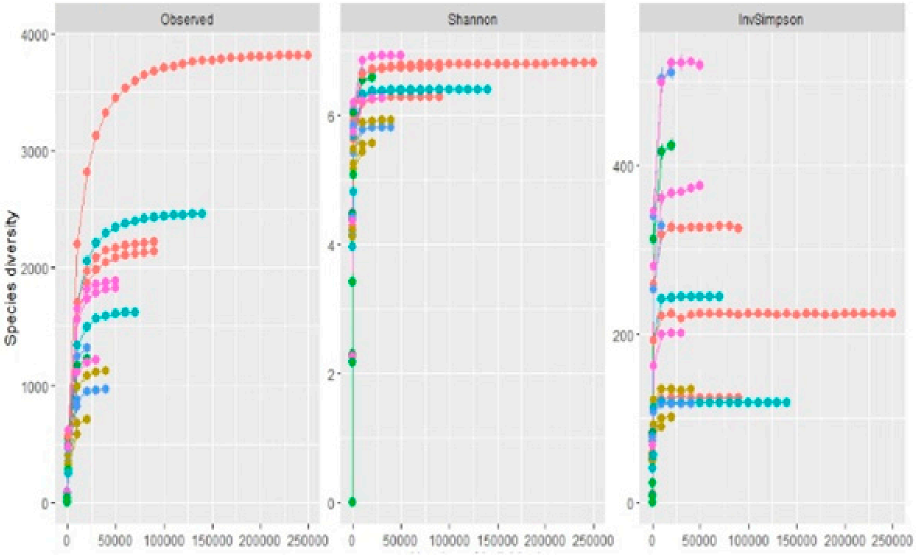

Shelf St4

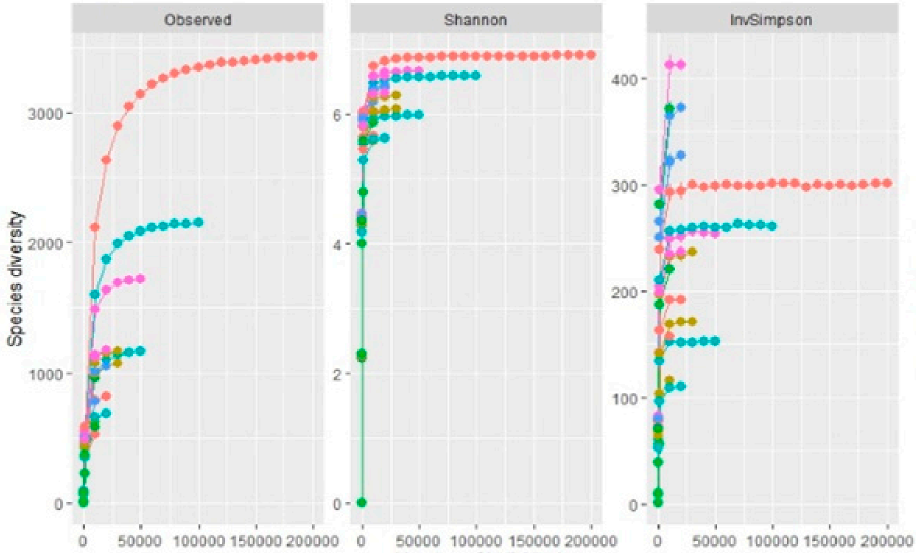

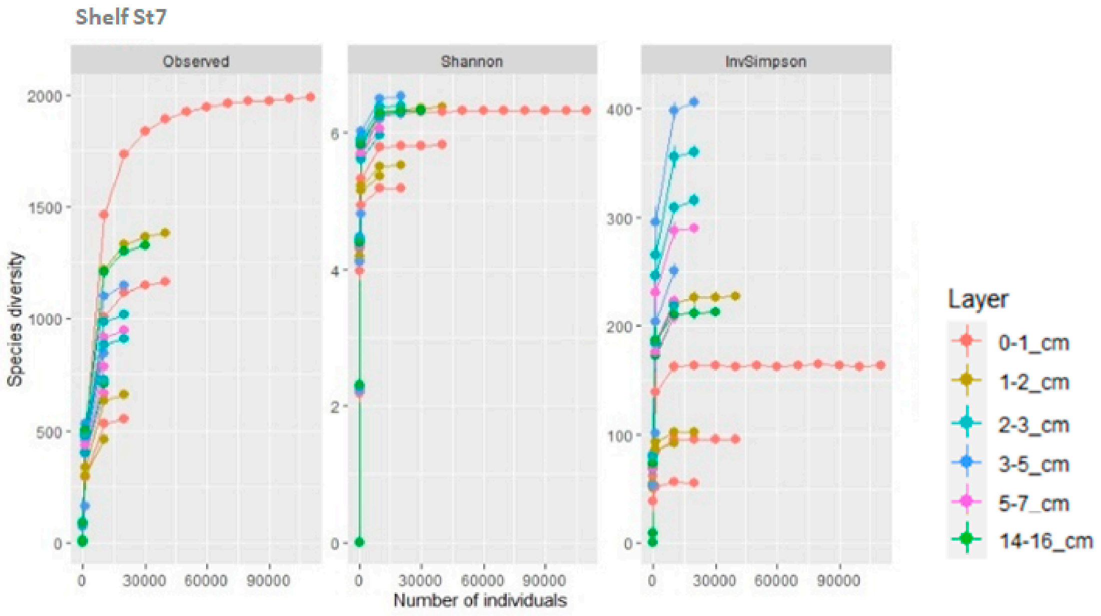

Figure S1: Diversity indices for bacterial communities in 6 different depths for 5 shelf stations across the eastern coast of Antarctic Peninsula.

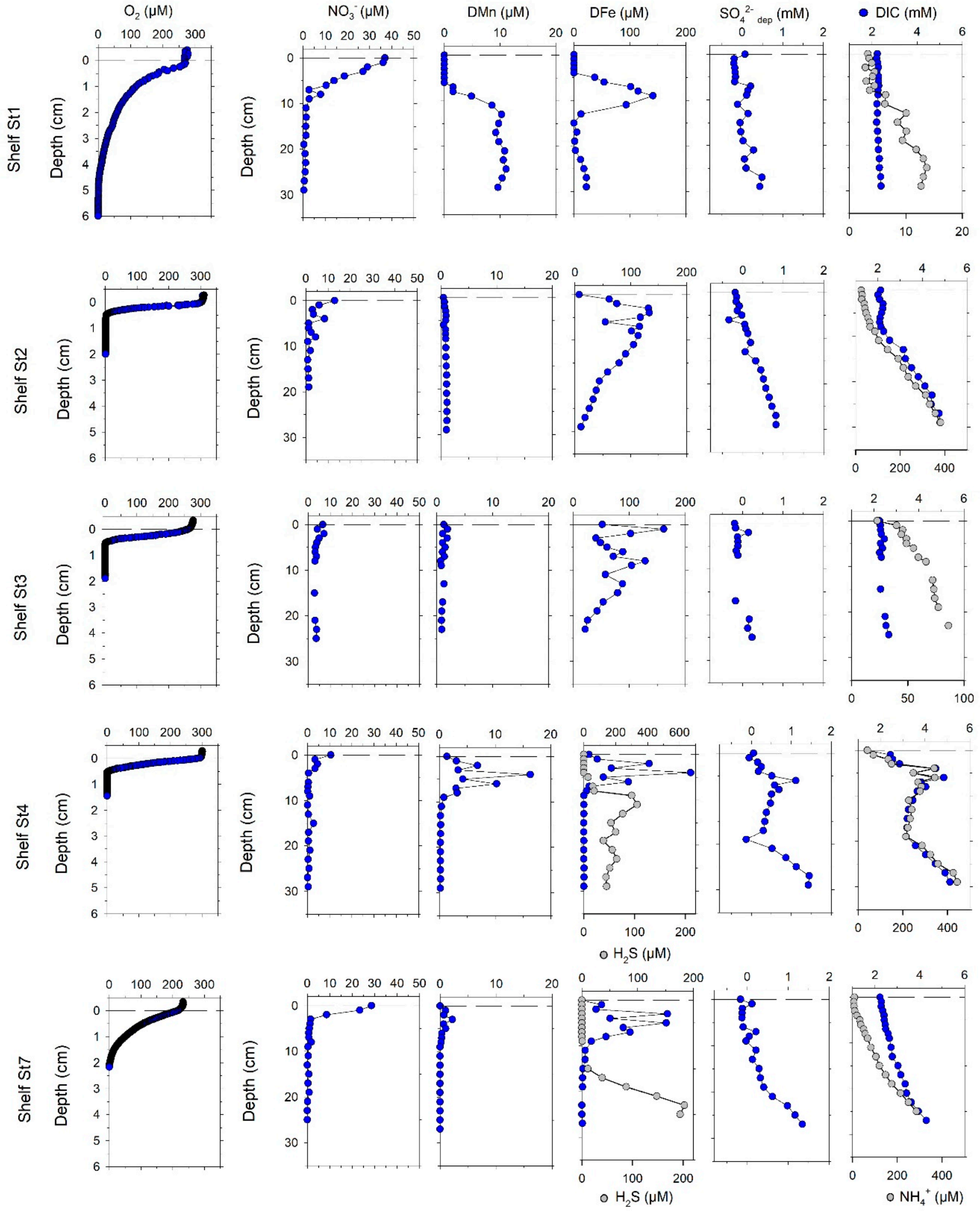

Figure S2: Representative profiles of reactive pore water compounds at the 5 shelf stations. $SO_4{}^{2-}$ depletion profiles calculated based on the molar ratio of Cl^- and $SO_4{}^{2-}$ of seawater [1]). Note that the scale for DFe and $NH_4{}^+$ concentrations changes between stations. Free H_2S was present only at Shelf St4 and Shelf St5. For further information: Baloza et al, [2].

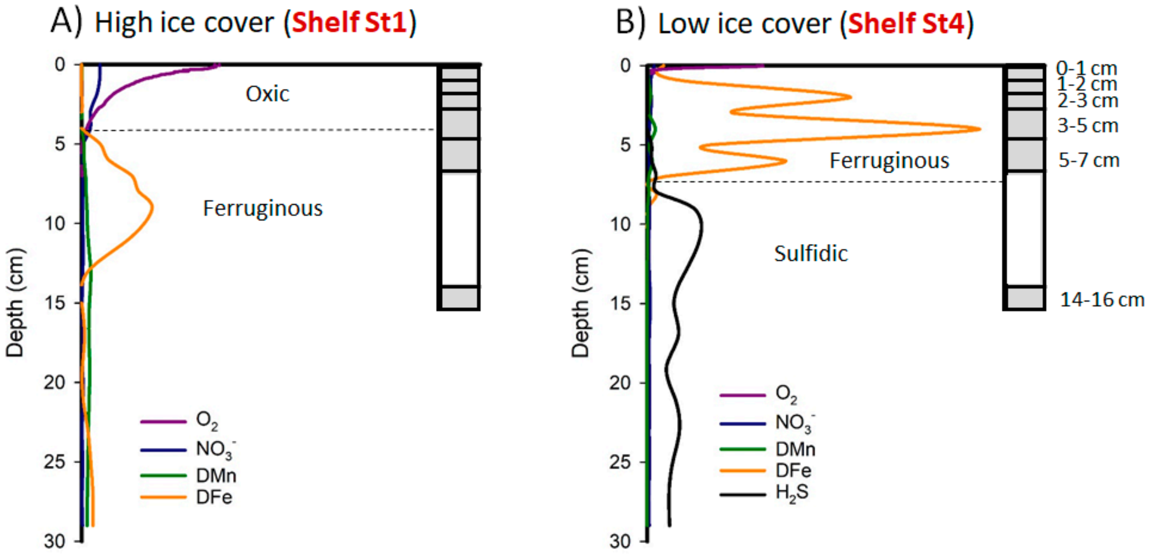

Figure S3: Schematic illustration of the porewater profiles that were typical for heavy ice cover (Shelf St1) (A) and low ice cover (Shelf St4) (B) of this study. The dashed lines mark the borders of the individual redox zones. The gray boxes to the right of each panel show the core sectioning schemes.

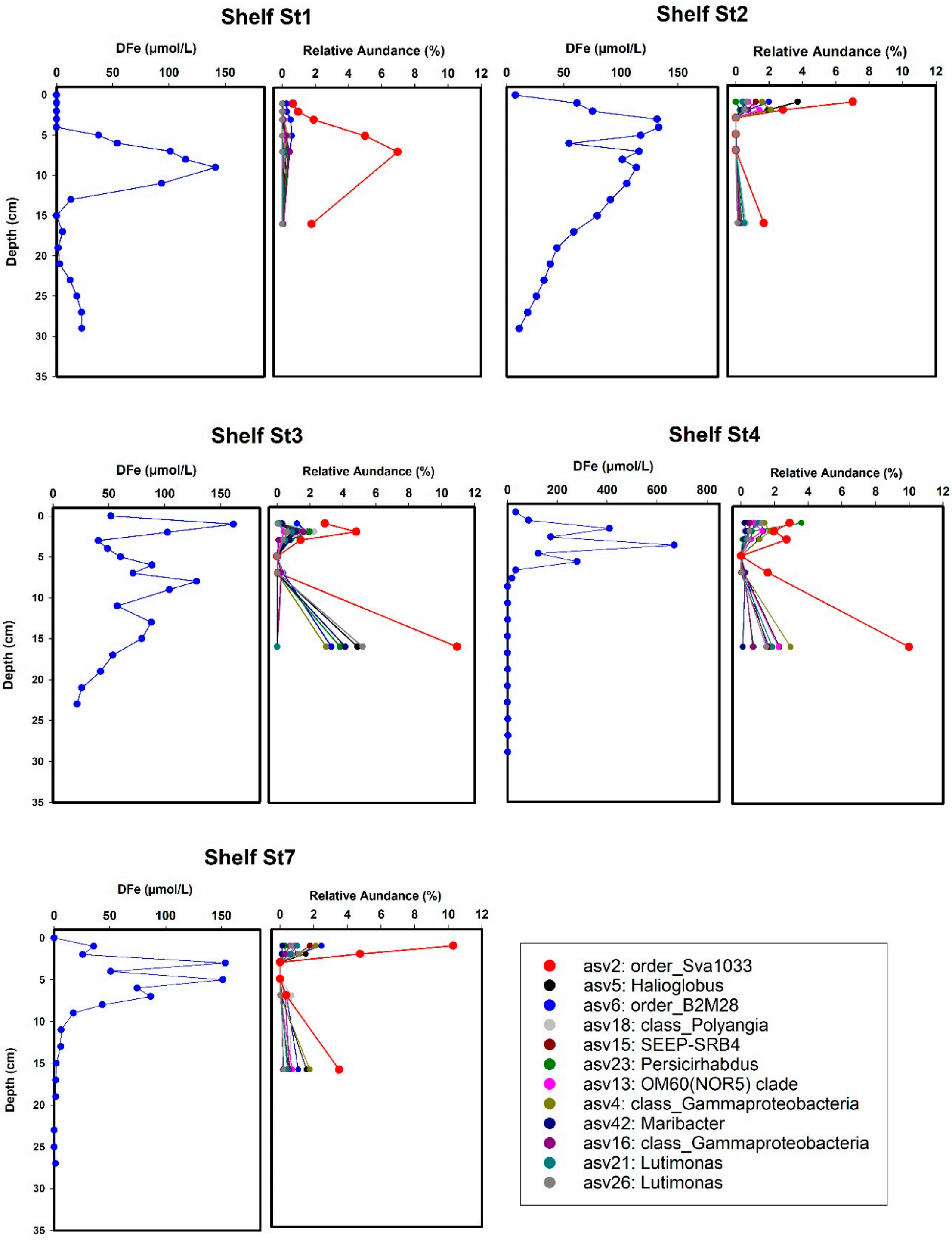

Figure S4: Representative profiles of the depth distribution of putative Fe-reducing bacteria in relation to dissolved/pore-water iron (DFe) concentrations. 12 different taxa were identified by applying differential abundance analysis. The relative abundance from one sediment core. DFe concentrations were measured in a core near (<1m) the collected microbial samples.

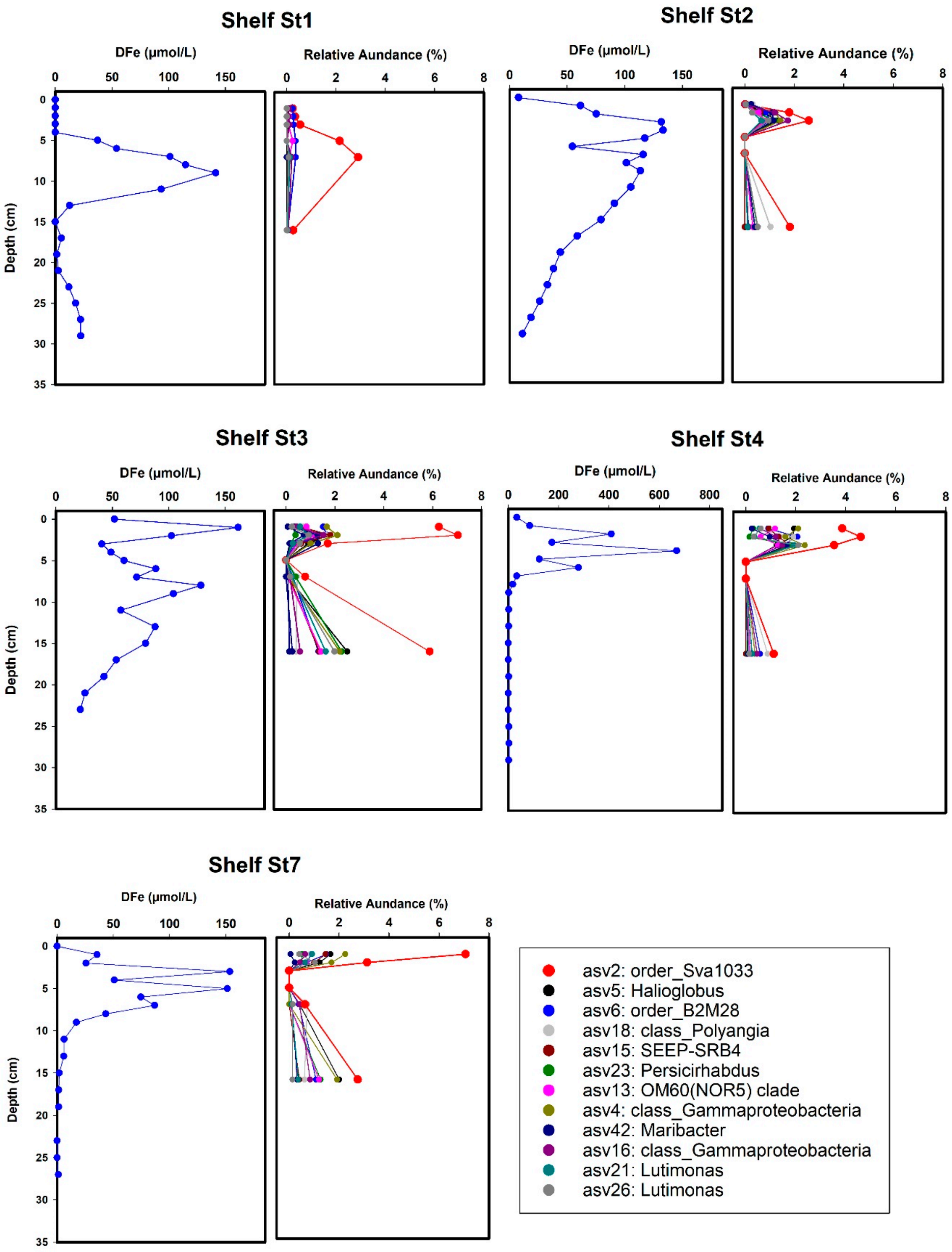

Figure S5: Representative profiles of the depth distribution of putative Fe-reducing bacteria in relation to dissolved/pore-water iron (DFe) concentrations. 12 different taxa were identified by applying differential abundance analysis. The relative abundance from one sediment core. DFe concentrations were measured in a core near (<1m) the collected microbial samples.

References

1. Weston, N.B.; Porubsky, W.P.; Samarkin, V.A.; Erickson, M.; Macavoy, S.E.; Joye, S.B. Porewater stoichiometry of terminal metabolic products, sulfate, and dissolved organic carbon and nitrogen in estuarine intertidal creek-bank sediments. *Biogeochemistry* **2006**, *77*, 375-408.
2. Baloza, M.; Henkel, S.; Geibert, W.; Kasten, S.; Holtappels, M. Benthic Carbon Remineralization and Iron Cycling in Relation to Sea Ice Cover Along the Eastern Continental Shelf of the Antarctic Peninsula. *Journal of Geophysical Research: Oceans* **2022**, *127*. https://doi.org/10.1029/2021JC018401.
